# Supplementary material for: Spin-polarized surface resonances accompanying topological surface state formation
Source: Nat Commun. 2016 Oct 14;7:13143. doi: 10.1038/ncomms13143 (PMC5067600; doi:10.1038/ncomms13143)
Supplement: Supplementary Information — Supplementary Figures 1-6 [file ncomms13143-s1.pdf]

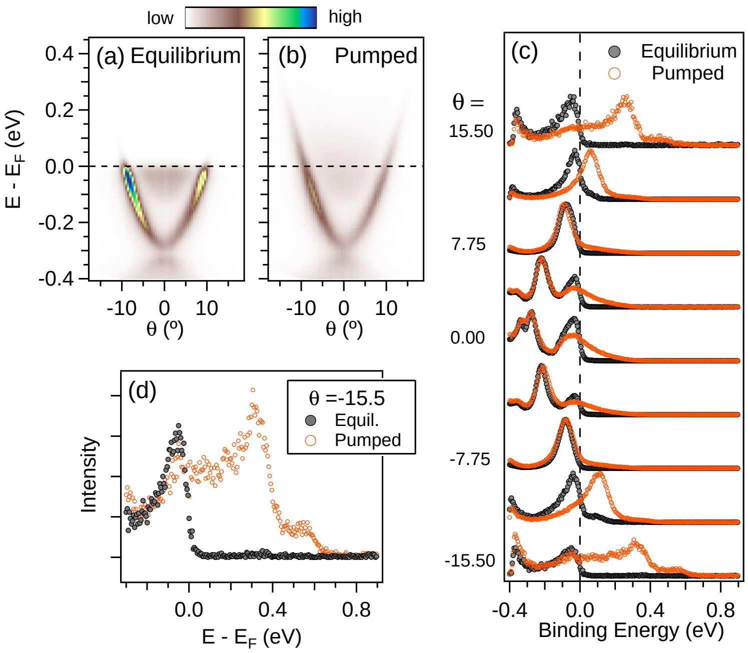

Supplementary Figure 1. Spectral signatures of the unoccupied surface resonance observed in time resolved photoemission measurements even without spin resolution. The equilibrium and pumped (Δt=0.7 ps) spectra along the Γ-K direction are shown in (a) and (b), respectively, on identical intensity colorscales. (c) Energy distribution curves for the equilibrium and pumped sample. It is possible to track the dispersion of the topological surface state well above the Fermi energy $E_{\rm F}$ due to the transient population created by the pump. (d) At higher emission angles a peak associated with the unoccupied surface resonance can be identified $\sim$550 meV above $E_{\rm F}$.

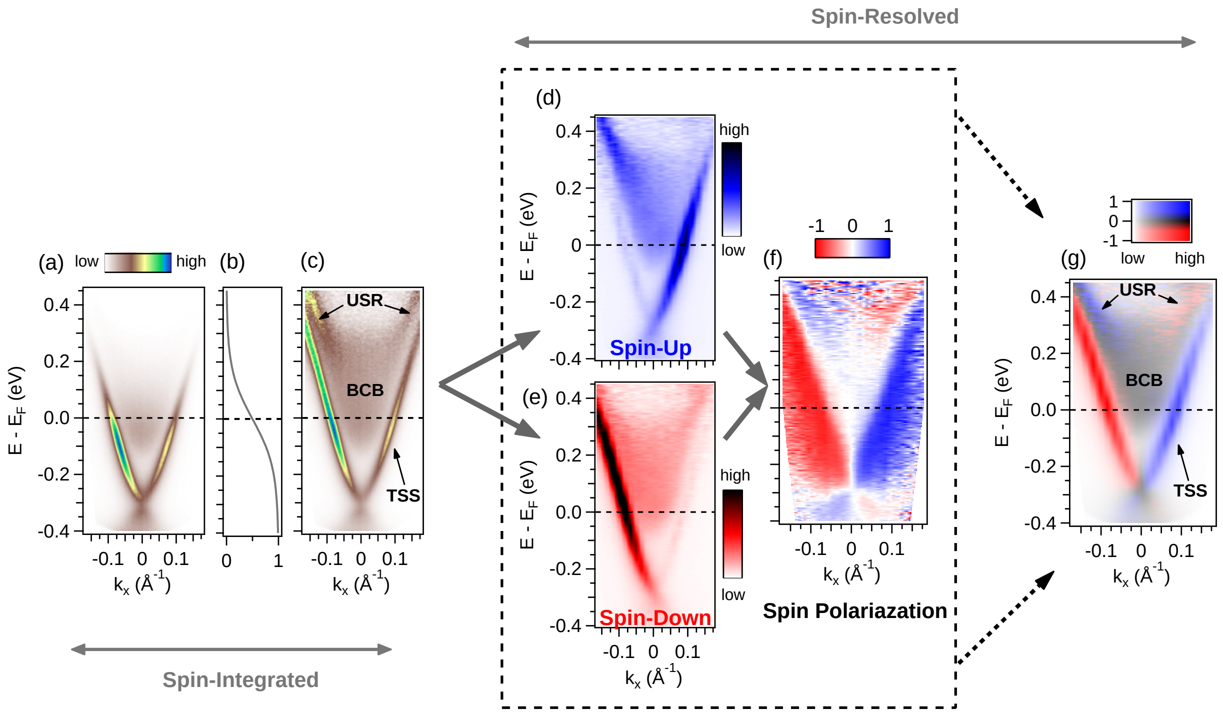

Supplementary Figure 2. Presentation methods of spin and time resolved photoemission data. (a) The raw data of an ARPES measurement on optically excited Bi_2Se_3 plotted on an intensity colorscale. Because it is spin-integrated, it consists of the sum of both spin channels ($I_\uparrow + I_\downarrow$). (b) The redistribution of carriers induced by the pump can be approximated by a Fermi Dirac distribution with elevated temperature $k_B T$=85 meV. (c) The spin-integrated spectrum divided by the effective Fermi Dirac distribution, allowing easier visualization of states above the Fermi energy E_F. (d) and (e) The intensities of the separate spin-up $I_\uparrow$ and spin-down $I_\downarrow$ channels. For visual clarity, both of these panels are divided by the effective Fermi Dirac distribution. (f) The spin-polarization ($P = (I_\uparrow - I_\downarrow) / (I_\uparrow + I_\downarrow)$) computed from these two channels. P contains no intensity information, and is therefore unaffected by the Fermi-Dirac division. Since it can take values from -1 to +1, it is suitably presented in a red-white-blue colorscale where unpolarized features appear white. (g) The full dataset (divided by the Fermi Dirac distribution) plotted with a two-dimensional colorscale. The spin-polarization is still conveyed through color (now red-black-blue, vertical axis of the colorscale), while the intensity information is encoded by the saturation (white = zero intensity, horizontal axis of the colorscale). The main advantage of this plotting method is that regions with low photoemission intensity are visually suppressed, so that the intrinsic positions and widths of spectral peaks are more accurately conveyed.

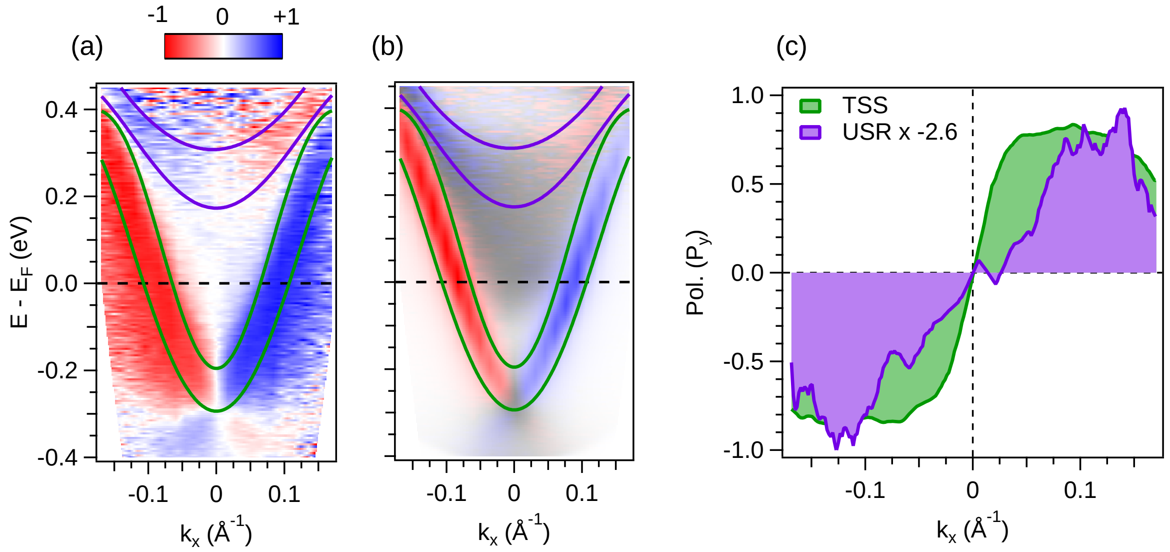

Supplementary Figure 3. Momentum-dependent analysis of the spin polarizations of the topological surface state and unoccupied surface resonance. The spin-resolved spectrum is plotted as (a) a polarization map and (b) with a two-dimensional colorscale to indicate both intensity and spin-polarization. The spectral regions of the topological surface state (TSS) and unoccupied surface resonance (USR) are indicated by green and purple curves, respectively. At each momentum within these windows, we integrate the spin-up and spin-down intensity along the energy axis, and use this to compute a momentum-dependent spin-polarization for each state. (c) The computed spin-polarization plotted vs k_x. The TSS exhibits a maximum polarization of ~ 0.85 and abruptly switches sign at $k_x = 0$. The width of this crossover is likely dominated by the finite momentum resolution of the experiment (~ 0.02 Å^{-1}) since the TSS is expected to maintain its polarization all the way to Γ. In contrast, the polarization of the USR exhibits a very gradual crossover from positive to negative values upon passing through $k_x = 0$, which suggests that its polarization is diminished in some finite momentum range around Γ. This is consistent with the DFT results and tight-binding calculations in the main text, which both indicate that the polarization of the TSS is maintained through Γ, while that of the USR vanishes in some range around Γ.

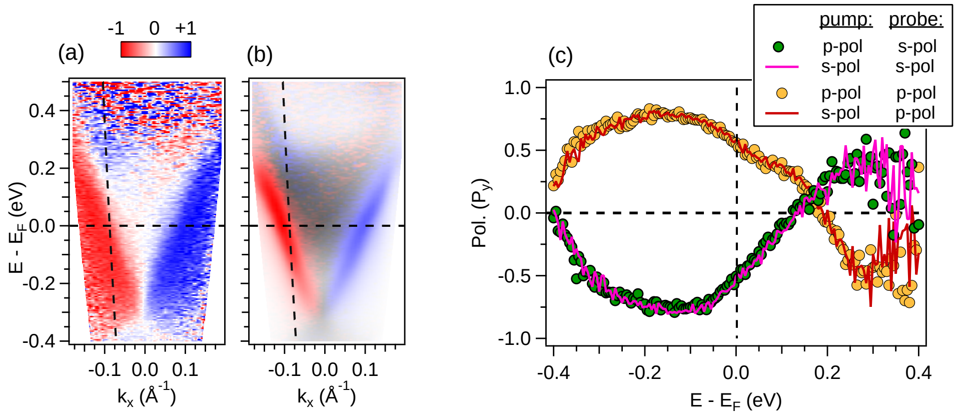

Supplementary Figure 4. Lack of pump polarization dependence on measured spin polarization. (a) The spin-polarization and (b) spin/intensity colormap along Γ-M with a *p*-polarized pump and *s*-polarized probe. For this figure, the crystal was rotated to align Γ-M to the lab k_x axis, with the spin-polarization measured along the orthogonal y-direction. (c) The measured spin polarization at the fixed emission angle indicated by vertical dashed lines in the previous panels. The spin-polarization reverses upon switching the probe polarization, but the pump polarization has no effect. This is consistent with the interpretation that the measured spin-polarizations are associated with polarized crystal eigenstates, rather than transient polarizations excited by spin-dependent matrix elements.

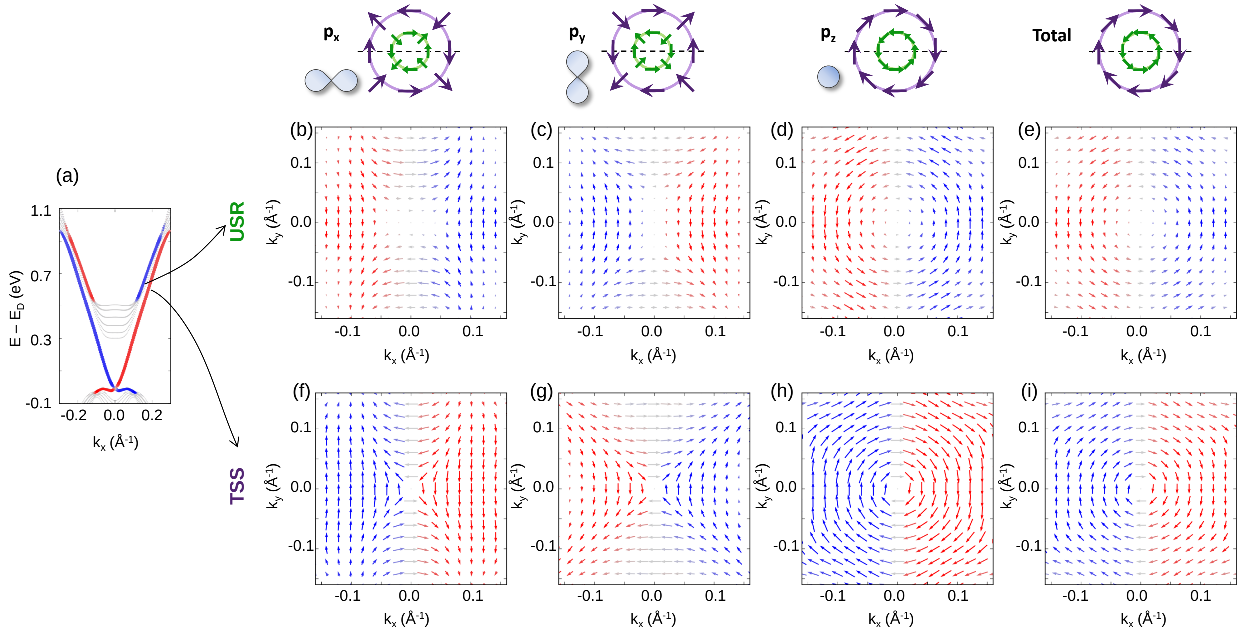

Supplementary Figure 5. Density functional theory calculations of the spin texture of Bi_2Se_3. In the main text we plot the DFT results along Γ-K only. Here we show the spin texture for the unoccupied surface resonance (USR) and topological surface state (TSS) along both Γ-K (k_x) and Γ-M (k_y). The length/direction of the arrows indicate the magnitude/orientation of the spin polarization, whereas the color denotes the y-component of the polarization. These calculations show that the TSS and USR have similar, yet oppositely oriented, spin textures. This striking anti-correlation is indicative of a deep relationship between the TSS and the USR, as elucidated in the main text.

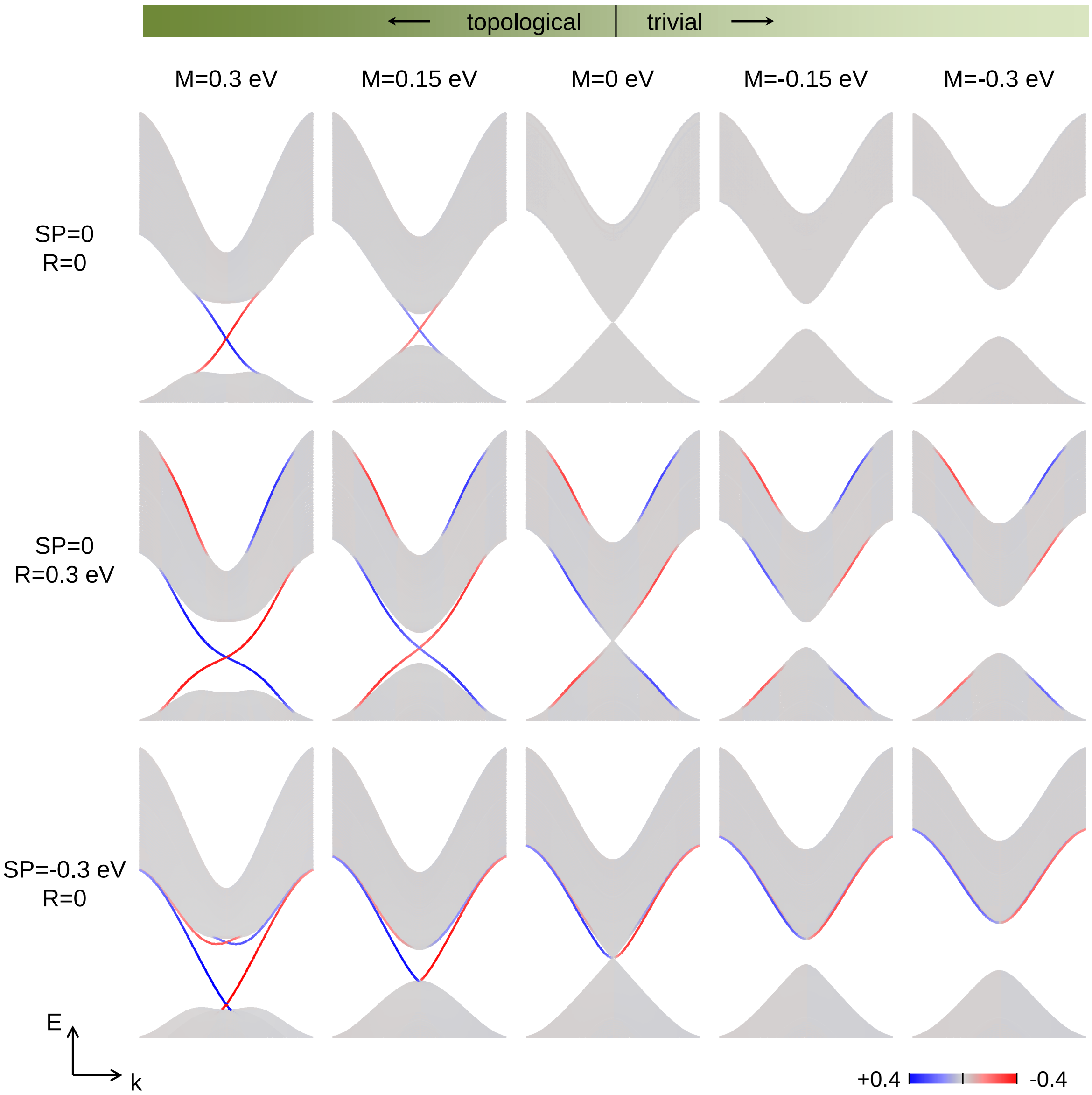

Supplementary Figure 6. Tight-binding calculations of Bi_2Se_3. The colors represent the magnitude of the y-component of the polarization. For the minimal case (top row), no spin-polarized or surface-localized features appear besides the topological surface state. The addition of a Rashba term (middle row) leads to the formation of spin-polarized surface resonances, as described in the main text. The electrostatic surface potential (third row) also leads to the formation of spin-polarized surface resonances, though their dispersion (below the bulk valence band) is qualitatively different from that measured experimentally. Nevertheless, the fact that even a simple electrostatic potential gives rise to spin-polarized surface resonances suggests that these states may be ubiquitous in broad classes of topological insulator materials.
